# Increased risk of sudden sensorineural hearing loss in patients with cervical spondylosis

**DOI:** 10.1038/s41598-024-52875-x

**Published:** 2024-02-05

**Authors:** Chia-Chun Liu, I-Te Chen, Shih-Feng Weng

**Affiliations:** 1 Department of Otorhinolaryngology, Yuan’s General Hospital, Kaohsiung, Taiwan; 2 Department of Healthcare Administration and Medical Informatics, Kaohsiung Medical University, 100, Shin-Chuan 1st Road, San Ming District, Kaohsiung, 80708 Taiwan; 3 Center for Medical Informatics and Statistics, Office of R&D, Kaohsiung Medical University, Kaohsiung, Taiwan; 4 Department of Medical Research, Kaohsiung Medical University Hospital, Kaohsiung, Taiwan

**Keywords:** Anatomy, Diseases, Health care, Medical research, Risk factors

## Abstract

Whether cervical spondylosis (CS) is a risk factor for sudden sensorineural hearing loss (SSNHL) remains unclear. This study used national population-based data to investigate the risk of SSNHL in patients with CS in Taiwan of different ages and sexes. This study used data covering 2 million people in Taiwan, which were obtained from the National Health Insurance Research Database. The data that support the findings of this study are available from National Health Insurance Research Database but restrictions apply to the availability of these data, which were used under license for the current study, and so are not publicly available. Data are however available from the corresponding authors upon reasonable request and with permission of National Health Insurance Research Database. This retrospective cohort study enrolled 91,587 patients with a newly diagnosed CS between January 2000 and December 2018. Case and control cohorts were matched 1:1 according to age, sex, and comorbidities. SSNHL incidence rate and risk were compared between the groups. Cox regression was used to estimate hazard ratios (HRs) and 95% confidence intervals (CIs). The mean follow-up period was 8.80 (SD = 4.12) and 8.24 (SD = 4.09) years in the CS and control cohorts, respectively. The incidence rate of SSNHL in the CS cohort (85.28 per 100 000 person-years) was 1.49-fold significantly higher than that in the non-CS cohort (57.13 per 100,000 person-years) (95% CI 1.32–1.68, *P* < .001). After age, sex, and selected comorbidities were adjusted for, CS exhibited an independent risk factor for SSNHL (adjusted HR = 1.52; 95% CI   1.34–1.71, *P* < .001). An age-stratified analysis in this study demonstrated a strong and highly significant association between CS and SSNHL in patients aged < 35 years (IRR = 2.28, 95% CI   1.18–4.39, *P* = .013). This large-scale Taiwanese-population-based retrospective study found that CS was associated with an increased risk of SSNHL. Acute hearing loss in patients with CS, particularly at a young age, should be carefully evaluated, and prompt treatment for SSNHL should be initiated.

## Introduction

Sudden sensorineural hearing loss (SSNHL) is an otologic emergency indicated by a loss greater than 30 dB over 3 continuous pure-tone frequencies occurring within 3 days. The postulated pathophysiology of SSNHL includes labyrinthine vascular compromise, labyrinthine viral infection, intracochlear membrane rupture, and autoimmune inner ear disease^1^. According to estimates in the United States, there are from 11 to 77 individuals of SSNHL per 100,000 persons per year^2^. The crude incidence of SSNHL in Taiwan was 9.76 per 100,000 persons per year on the basis of a 14-year (from 2000 to 2013) nationwide study^3^. Lower incidence of SSNHL in Taiwan as compared with the United States may be due to different etiologic factors in different geographic regions or varied medical behavior of residents.

Cervical spondylosis (CS), also known as cervical osteoarthritis, is associated with degenerative osseocartilaginous components of the cervical spine. Intervertebral disc degeneration and abnormal osteophyte growth may result in painful neck stiffness and cervical myelopathy. Cervical spondylosis with arthritic osteophytes may produce extramural mechanical compression to the vertebral artery, particularly when the neck is rotated or extended, resulting in vertebrobasilar insufficiency^4–6^, which increases the risk of SSNHL because of the reduced labyrinthine arterial blood supply to the inner ear^7^. Cervical spondylosis (CS) was estimated to be prevalent in > 50% of the population aged over 40 years^8^. CS is more common in male than in female and is with a peak incidence between the ages of 40 and 60 years for both gender groups^9^.

According to our review of the literature, no previous study has investigated the association between CS and SSNHL in a population-based cohort. Therefore, to obtain sufficient statistical power, we used a large-scale population-based cohort and investigated the association between CS and SSNHL.

## Patients and methods

### Data sources

Taiwan’s single-payer National Health Insurance (NHI) program was established on March 1, 1995. The NHI contains health insurance claims data for 99% of Taiwan’s 23 million residents^10^. The NHI program is one of the world’s largest and most integrated population-based datasets. The National Health Insurance Research Database (NHIRD) contains integrated information on prescription details, clinic visits, surgical procedures, registry of catastrophic illness, and medical expenditures. Disease diagnoses were coded according to the *International Classification of Diseases, 9th and 10th Revision, Clinical Modification* (*ICD-9-CM* and *ICD-10-CM*). The personal information of all patients included in the NHIRD is encrypted to ensure their privacy. Therefore, the requirement for written informed consent was waived.

This retrospective cohort studied a sample of data from the NHIRD covering 2 million systemic beneficiaries randomly selected in 2005 and their medical claims data (from 2000 to 2018). According to the Taiwan National Health Research Institute, no significant differences were observed in sex distribution, age distribution, or average insured payroll-related amount among patients enrolled in the NHIRD^11^. The manuscript is reported following the STROBE checklist of observational studies. (Supplementary Information).

This study was approved by the Institutional Review Board of Yuan’s General Hospital, Kaohsiung, Taiwan (IRB No. 20220307B).

### Study design and patients

We identified patients aged ≥ 18 years with newly diagnosed CS with *ICD-9* codes 721.0 and 721.1 and *ICD-10* codes M47.012, M47.022, M47.12, M47.22, and M47.812 from January 1, 2000, to December 31, 2018. We only included patients with CS diagnosed by an orthopedist or a neurosurgeon in ambulatory care claims or one-time hospitalization. These criteria improved the accuracy of the diagnosis of CS. Furthermore, we excluded patients who had sudden hearing loss prior to the diagnosis of CS. Patients diagnosed with Meniere's disease and acoustic neuroma before the CS diagnosis were also excluded.

To form the control cohort, patients were selected from the 2 million sub-datasets without CS and were matched with the case cohort according to age (± 30 days) and sex. The initial diagnosis of CS during the study period was defined as the index date, and the index date for the matched control was established by matching the index date of the patient with CS. We excluded controls with a diagnosis of sudden sensorineural hearing loss before the index date. In addition, we excluded patients diagnosed with Meniere’s disease and acoustic neuroma before the index date in both CS and control cohorts. Subsequently, case and control cohorts were matched 1:1 using propensity score (PS) according to age, sex, index date, hypertension (HTN), coronary artery disease (CAD), diabetes mellitus (DM), chronic renal disease, hyperlipidemia, and stroke. PS matching was used to reduce selection bias because observational study design may have various confounding. PS was calculated using a logistic regression model to estimate the risk of CS diagnosis, and the aforementioned confounding variables were used as independent variables. The SAS matching macro named “% OneToManyMTCH,” which was proposed during the 29th SAS Users Group International proceedings, was used in this study. Subsequently, 91,587 patients with CS and 91,587 matched control cohort were recruited in this study.

### Outcome and covariate measurements

Both case and control cohorts were followed until the diagnosis of SSNHL, death, withdrawal from the NHI program, or until the end of our study period (December 31, 2018). The primary outcome was SSNHL (*ICD-9-CM* code 388.2, *ICD-10-CM* codes H91.21, H91.22, H91.23). The diagnosis was made by an otorhinolaryngologist in either 2 ambulatory care claims or one-time hospitalization to increase the diagnostic accuracy of SSNHL^12^.

The binary variables were comorbidities associated with SSNHL, including hypertension (*ICD-9-CM* codes 401–405, *ICD-10-CM* codes I10-I15), CAD (*ICD-9-CM* codes 410–414, *ICD-10-CM* codes I20-I25), DM (*ICD-9-CM* code 250, *ICD-10-CM* codes E10-E14), chronic renal disease (*ICD-9-CM* codes 582, 583, 585, 586, and 588, *ICD-10-CM* codes N18-N19), hyperlipidemia (*ICD-9-CM* codes 272.0–272.4, *ICD-10-CM* code E78), and stroke (*ICD-9-CM* codes 430–438, *ICD-10-CM* codes I60-I66). These comorbidities were included if they were present either in hospitalization care claims within 1 year before the index date or were recorded in 3 or more ambulatory care claims.

### Statistical analysis

All analyses were performed using SAS software, version 9.4 (Cary, NC, USA). Patient basic demographic characteristics and comorbidities were compared between the CS and control groups using Pearson chi-square analysis for categorical variables and Student *t* test for continuous variables. The SSNHL incidence was calculated as the total number of SSNHL patients discovered during the follow-up period, divided by the whole person-years for the respective groups according to age, sex, and comorbidities. Poisson regression analysis was performed to obtain the incidence rate ratio (IRR) to compare the risk of SSNHL between CS group and non-CS controls. Cox proportional hazards regression was used to determine the differences in the adjusted hazard ratios (HRs) and 95% confidence intervals (CIs) for the risk of SSNHL. The Kaplan–Meier method was used to construct the cumulative incidence curves, and the log-rank test was used to evaluate the differences. *P* < 0.05 indicated statistical significance.

### Ethics approval and consent to participate

This study was conducted according to the Declaration of Helsinki and was approved and granted exemption from review by the Institutional Review Board of Yuan’s General Hospital, Kaohsiung, Taiwan (IRB No. 20220307B). The need for informed consent was waived because the dataset analyzed in this study was devoid of any identifiable personal information.

## Results

A total of 91,587 patients with CS and 91,587 non-CS individuals were matched for analysis. Both groups’ baseline demographic characteristics and comorbidities are listed in Table 1. No significant differences in age, sex, and baseline comorbidities were found between the 2 groups. Approximately 39% of all participants were between 50 and 64 years old, and approximately 56% of both cohorts were women. The mean follow-up period was 8.80 (standard deviation [SD], 4.12) years for the CS group and 8.24 (SD, 4.09) years for the non-CS group.

**Table 1:** Comparisons in demographic characteristics and comorbidities in patient with and without cervical spondylosis.

	Cervical spondylosis		p-value
	Yes	No	
	(N = 91,587)	(N = 91,587)	
Sex			
Male	40,016 (43.69)	40,016 (43.69)	1.00
Female	51,571 (56.31)	51,571 (56.31)	
Age (years; mean ± SD)	53.39 ± 13.90	53.39 ± 13.90	1.00
Age group (years)	Number (%)	Number (%)	
0–34	7949 (8.68)	7949 (8.68)	1.00
35–49	28,347 (30.95)	28,347 (30.95)	
50–64	35,768 (39.05)	35,768 (39.05)	
≥ 65	19,523 (21.32)	19,523 (21.32)	
Baseline comorbidity			
HTN	28,551 (31.17)	28,625 (31.25)	0.71
CAD	14,069 (15.36)	14,068 (15.36)	0.99
DM	13,293 (14.51)	13,278 (14.5)	0.92
Chronic renal disease	3905 (4.26)	3842 (4.19)	0.46
Hyperlipidemia	21,919 (23.93)	21,923 (23.94)	0.98
Stroke	7158 (7.82)	7149 (7.81)	0.94
Follow-up period (years; mean ± SD)	8.80 ± 4.12	8.24 ± 4.09	< 0.001
Medical expenditure (NTD/year)	370,858.24 ± 788,717.67	295,523.88 ± 792,397.08	< 0.001
OPD visit (times)	207.76 ± 193.94	133.14 ± 148.05	< 0.001
Admission (times)	2.04 ± 4.50	1.66 ± 4.61	< 0.001

The demographic characteristics and comorbidities between the CS and control groups were compared using Pearson chi-square tests. Numbers in parentheses represent percentages.

*SSNHL* sudden sensorineural hearing loss, *HTN* hypertension, *CAD* coronary artery disease, *DM* diabetes mellitus, *SD* standard deviation.

During the follow-up period, a higher incidence rate of SSNHL was observed in patients with CS (85.28 per 100 000 person-years) than that in the non-CS matched group (57.13 per 100 000 person-years), leading to a significant difference in the IRR of SSNHL (1.49, 95% CI 1.32–1.68; Table 2). The IRR stratified by age revealed a significantly higher number of the patients with CS than the patients without CS in all 4 age groups. Furthermore, the patients with CS with aged < 35 years exhibited the highest IRR of 2.28 (95% CI 1.18–4.39, *P* = 0.013), followed by patients aged 50–64 years with IRR of 1.60 (95% CI 1.34–1.92, *P* < 0.001), patients aged ≥ 65 years with IRR of 1.44 (95% CI 1.13–1.83, *P* = 0.003), and patients aged 35–49 years with IRR of 1.36 (95% CI 1.08–1.72, *P* = 0.009), as shown in Table 2. Men and women with CS had a significantly higher IRR of SSNHL than matched controls. The IRR for men and women was 1.61 (95% CI 1.36–1.89, *P* < 0.001) and 1.37 (95% CI 1.15–1.63, *P* < 0.001), respectively (Table 2). In addition, we found that compared with the controls, patients with CS consistently had a higher IRR of stroke (IRR: 1.58, 95% CI 1.04–2.40, *P* = 0.03), DM (IRR: 1.39, 95% CI 1.05–1.83, *P* = 0.02), hyperlipidemia (IRR: 1.27, 95% CI 1.01–1.59, *P* = 0.04), and hypertension (IRR: 1.25, 95% CI 1.03–1.52, *P* = 0.03), as shown in Table 2.

**Table 2:** Risk of sudden sensorineural hearing loss (SSNHL) in the cervical spondylosis and control groups.

Characteristics	Cervical spondylosis				Controls				IRR (95%CI)	p-value
	N	SSNHL	PY	Rate*	N	SSNHL	PY	Rate*		
All	91,587	687	805,612.77	85.28	91,587	431	754,485.76	57.13	1.49 (1.32–1.68)	< 0.001
Sex										
Male	40,016	388	456,818.96	84.94	40,016	225	425,702.81	52.85	1.61 (1.36–1.89)	< 0.001
Female	51,571	299	348,793.81	85.72	51,571	206	328,782.95	62.66	1.37 (1.15–1.63)	< 0.001
Age (years)										
0–34	7949	35	73,041.5	47.92	7949	12	57,108.97	21.01	2.28 (1.18–4.39)	0.013
35–49	28,347	183	255,965.8	71.49	28,347	117	222,795.96	52.51	1.36 (1.08–1.72)	0.009
50–64	35,768	315	305,529.52	103.10	35,768	187	291,051.08	64.25	1.60 (1.34–1.92)	< 0.001
≥ 65	19,523	154	171,075.96	90.02	19,523	115	183,529.75	62.66	1.44 (1.13–1.83)	0.003
Comorbidities										
HTN	28,551	225	239,112.96	94.10	28,625	180	238,880.55	75.35	1.25 (1.03–1.52)	0.03
CAD	14,069	122	118,442.53	103.00	14,068	96	119,301.34	80.47	1.28 (0.98–1.67)	0.07
DM	13,293	120	107,211.4	111.93	13,278	87	108,037.92	80.53	1.39 (1.05–1.83)	0.02
Chronic renal disease	3905	31	29,974.49	103.42	3842	22	30,628.57	71.83	1.44 (0.83–2.49)	0.19
Hyperlipidemia	21,919	174	166,221.77	104.68	21,923	135	163,656.05	82.49	1.27 (1.014–1.59)	0.04
Stroke	7158	55	59,974.82	91.705	7149	36	61,830.72	58.22	1.58 (1.035–2.40)	0.03

A Poisson regression analysis was performed to calculate the incidence rate ratio. *SSNHL* sudden sensorineural hearing loss, *IRR* incidence rate ratio, *PY* person-years, Rate*: per 100,000 person-years, *HTN* hypertension, *CAD* coronary artery disease, *DM* diabetes mellitus.

**Figure 1:**
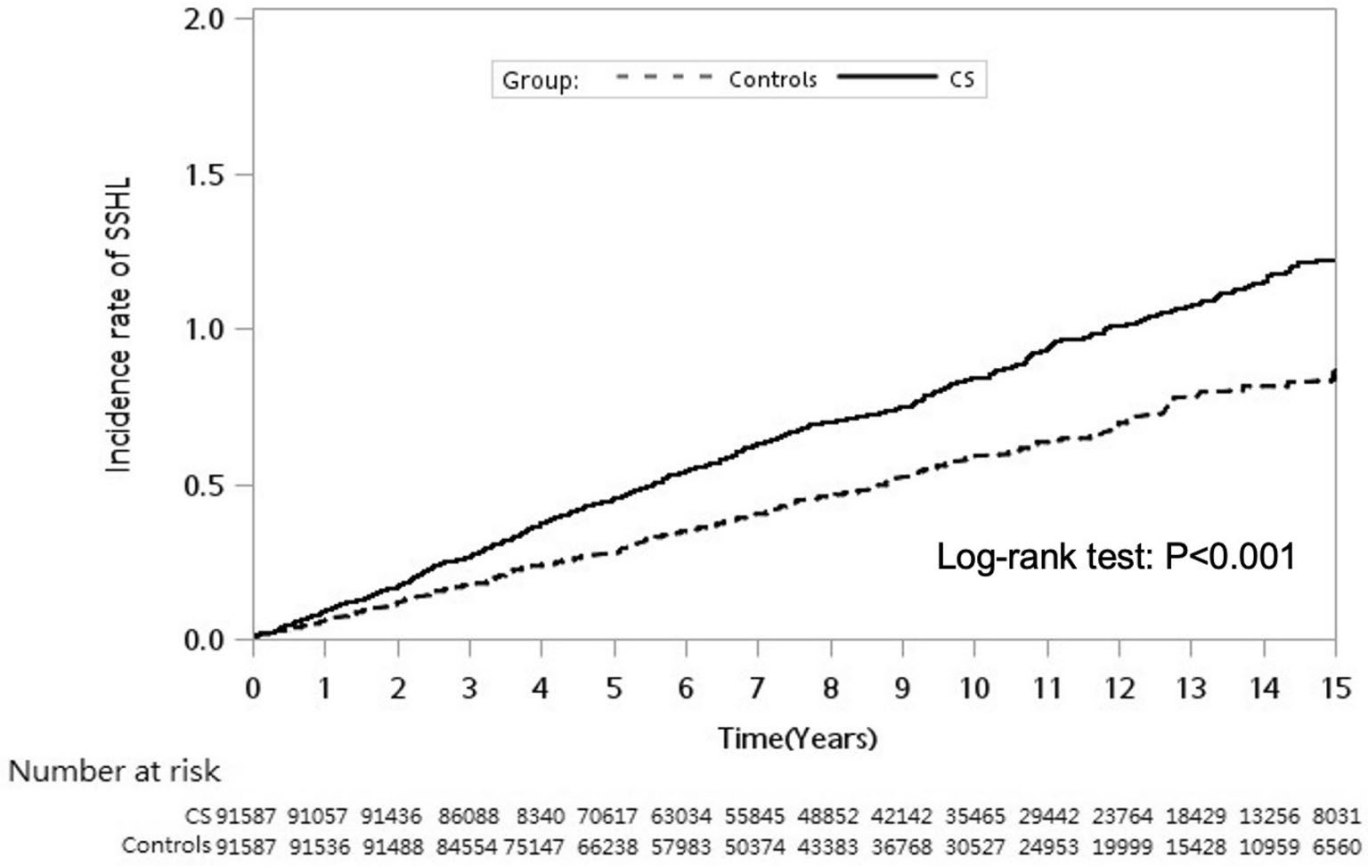
Cumulative incidence rate of sudden sensorineural hearing loss (SSNHL) in patients with cervical spondylosis (CS) and controls during the follow-up period.

Table 3 presents the crude and adjusted HRs for SSNHL during the follow-up period. After age, sex, and the selected comorbidities were adjusted for, the CS cohort had an independent risk of SSNHL (adjusted HR = 1.52; 95% CI 1.34–1.71; *P* < 0.001). Furthermore, compared with patients aged 0–34 years, patients aged > 34 years had a significantly higher risk of SSNHL; patients aged 35–49 years (adjusted HR = 1.70; 95% CI   1.25–2.32; *P* < 0.001), 50–64 years (adjusted HR = 2.15; 95% CI 1.59–2.91; *P* < 0.001), and ≥ 65 years (adjusted HR = 1.83; 95% CI   1.32–2.54; *P* < 0.001). However, hyperlipidemia was an independent risk factor for SSNHL (adjusted HR = 1.20; 95% CI   1.03–1.40; *P* = 0.02) but not sex or comorbidities. The Kaplan–Meier analysis showed that the cumulative incidence of SSNHL was significantly higher in patients with CS than that in individuals without CS, and the findings of the log-rank test were also significant (*P* < 0.001; Fig. 1).

**Table 3:** Crude and adjusted hazard ratios for the Cox proportional hazard regression analysis and 95% confidence intervals for sudden sensorineural hearing loss in the study cohort during the follow-up period.

Cohort	Crude hazard ratio (95% CI)	p-value	Adjusted hazard ratio (95% CI)	p-value
Cervical spondylosis				
Yes	1.50 (1.33–1.69)	< 0.001	1.52 (1.34–1.71)	< 0.001
No	1.00		1.00	
Sex				
Male	1.07 (0.95–1.21)	0.24	1.08 (0.96–1.22)	0.20
Female	1.00		1.00	
Age (years)				
0–34	1.00		1.00	
35–49	1.74 (1.28–2.36)	< 0.001	1.70 (1.25–2.32)	< 0.001
50–64	2.33 (1.73–3.14)	< 0.001	2.15 (1.59–2.91)	< 0.001
≥ 65	2.11 (1.55–2.87)	< 0.001	1.83 (1.32–2.54)	< 0.001
Comorbidity				
HTN	1.28 (1.14–1.45)	< 0.001	1.05 (0.90–1.22)	0.54
CAD	1.35 (1.16–1.56)	< 0.001	1.15 (0.97–1.37)	0.10
DM	1.42 (1.22–1.65)	< 0.001	1.18 (1.00–1.40)	0.06
Chronic renal disease	1.23 (0.93–1.62)	0.14	1.03 (0.77–1.36)	0.86
Hyperlipidemia	1.42 (1.25–1.62)	< 0.001	1.20 (1.03–1.40)	0.02
Stroke	1.05 (0.85–1.30)	0.68	0.87 (0.69–1.09)	0.21

The adjusted hazard ratio for developing sensorineural hearing loss was calculated using the Cox proportional hazard regression analysis. *CI* confidence interval, *HTN* hypertension, *CAD* coronary artery disease, *DM* diabetes mellitus.

## Discussion

According to our review of the literature, this study is the biggest large-scale retrospective population-based study to examine the risk of SSNHL in a national cohort of Asian patients with CS. We investigated 91,587 patients with CS and 91,587 controls. In addition, we demonstrated that the patients with CS had a higher incidence of SSNHL than the controls. The relative risk of developing SSNHL in patients with CS increased 1.52 times in the whole cohort after adjustment for age, sex, hypertension, CAD, DM, chronic renal disease, hyperlipidemia, and stroke. The log-rank test showed a significantly higher incidence rate of SSNHL in patients with CS than that in the comparison cohort.

The etiology of SSNHL is multifactorial, and the proposed mechanism involves infection, autoimmune conditions, vascular insufficiency, and rupture of the labyrinthine membrane^13–15^. Several studies have proposed that vascular events, including thromboembolism, altered blood circulation, and vasospasm, may be associated with a higher risk of SSNHL^16–18^. The labyrinthine artery, which originates from the vertebrobasilar system of posterior brain circulation, serves as the primary source of arterial supply to the inner ear. Other studies have found an association between the hemodynamic instability of the vertebrobasilar system and SSNHL^19,20^. Hsu et al. used the NHIRD^5^ and found that patients with vertebrobasilar insufficiency have an increased risk of developing SSNHL.

The association between CS and vertebrobasilar insufficiency has been elucidated in some studies^5,21,22^. Osteophytes formed at the uncinate process of the vertebrae may cause compromised blood flow in the vertebral artery^23,24^. In addition to anatomic compression, cervical sympathetic trunk irritation caused by CS is another mechanism of vasoconstriction, which was proposed by Liu et al.^25^. We speculated an association between CS and SSNHL. The sample of this retrospective cohort study was relatively large. The findings showed that the risk for SSNHL is higher in patients with CS than in the general population, even after confounders were adjusted for. The sex-stratified analysis showed that both men and women with CS had a significantly higher risk of SSNHL than the patients without CS (Table 2). In addition, older patients exhibited an increased risk of SSNHL (Table 3). This may be explained by a higher incidence of vascular insults or ischemic events in older patients, which was found by Wu et al.^26^. According to a 14-year nationwide population-based study on the epidemiologic incidence in patients with sudden sensorineural hearing loss by CY Kuo et al.^3^, in which total, 31,258 patients were included from 2000 to 2013 in Taiwan. The patients most commonly presented with SSNHL were observed in the age group of 45–64 years, which appealed much identical to our finding in the different age groups. We may explain the result as the passive medical behavior in elder patients in Taiwan, singly decreased hearing level without acute distress like vertigo or otalgia may be neglected. In addition, immobility to seek medical assistance and other comorbidities delay the timely diagnosis and record in the National Health Insurance Research Database. Despite the higher occurrence of SSNHL associated with advanced age, our attention should be directed towards younger patients with CS, especially those aged < 35 years. This emphasis on the younger age group is rooted in our observation of a notably higher Incidence Rate Ratio (IRR) of SSNHL when comparing CS patients to age-matched controls, as detailed in our age-stratified analysis (Table 2). Patients aged < 35 years are generally relatively healthy having fewer comorbidities. Therefore, the effect of CS may play a major role in increasing the risk of SSNHL on patients aged < 35 years. According to recent studies on hearing recovery of SSNHL^27,28^, younger age and early initiation of clinical steroid treatment were independent predictors with a fair prognosis of SSNHL. Cautious and precise diagnosis of SSNHL in younger patients with CS may prevent permanent sensorineural hearing loss.

This study has some limitations. First, because the severity of CS was not indicated in the database, we could not determine whether the severity of CS influences the risk of SSNHL. Second, the population-based study has inherent limitations in exploring the pathophysiological mechanism of an association between CS and SSNHL. Finally, because the diagnoses of CS, SSNHL, and other diseases were coded according to *ICD-9*-*CM* and *ICD-10*-*CM* codes, misclassification by clinicians might be present.

## Conclusion

In conclusion, this large-scale Taiwanese-population-based study found that CS was associated with an increased risk of SSNHL. Our findings corroborate findings that CS is an independent risk factor even after confounders are adjusted for in a total cohort. These results suggest that clinicians should pay more attention to patients with CS with acute hearing loss, particularly younger patients.

### Supplementary Information

Supplementary Information.

## Data Availability

The data that support the findings of this study are available from National Health Insurance Research Database but restrictions apply to the availability of these data, which were used under license for the current study, and so are not publicly available. Data are however available from the corresponding authors upon reasonable request and with permission of National Health Insurance Research Database.
